# Screening NK-, B- and T-cell phenotype and function in patients suffering from Chronic Fatigue Syndrome

**DOI:** 10.1186/1479-5876-11-68

**Published:** 2013-03-20

**Authors:** Marta Curriu, Jorge Carrillo, Marta Massanella, Josepa Rigau, José Alegre, Jordi Puig, Ana M Garcia-Quintana, Jesus Castro-Marrero, Eugènia Negredo, Bonaventura Clotet, Cecilia Cabrera, Julià Blanco

**Affiliations:** 1 Institut de recerca de la sida, IrsiCaixa-HIVACAT, Institut d’Investigació en Ciències de la Salut Germans Trias I Pujol|, Badalona, Spain; 2 CFS Clinic, Tarragona, Spain; 3 CFS Unit, Institut de Recerca Vall d’Hebron, Barcelona, Spain; 4 Fundació Lluita contra la SIDA, Hospital Germans Trias I Pujol, Badalona, Spain; 5 CFS Unit, Delfos Clinic, Barcelona, Spain; 6 Institut de Recerca de la sida, IrsiCaixa/Institut d’Investigació en Ciències de la Salut Germans Trias i Pujol, Hospital Universitari Germans Trias i Pujol, Badalona, 08916, Spain

**Keywords:** T regulatory cells, NKp46, Immune activation, Immunosenescence

## Abstract

**Background:**

Chronic Fatigue Syndrome (CFS) is a debilitating neuro-immune disorder of unknown etiology diagnosed by an array of clinical manifestations. Although several immunological abnormalities have been described in CFS, their heterogeneity has limited diagnostic applicability.

**Methods:**

Immunological features of CFS were screened in 22 CFS diagnosed individuals fulfilling Fukuda criteria and 30 control healthy individuals. Peripheral blood T, B and NK cell function and phenotype were analyzed by flow cytometry in both groups.

**Results:**

CFS diagnosed individuals showed similar absolute numbers of T, B and NK cells, with minor differences in the percentage of CD4^+^ and CD8^+^ T cells. B cells showed similar subset frequencies and proliferative responses between groups. Conversely, significant differences were observed in T cell subsets. CFS individuals showed increased levels of T regulatory cells (CD25^+^/FOXP3^+^) CD4 T cells, and lower proliferative responses *in vitro* and *in vivo*. Moreover, CD8 T cells from the CFS group showed significantly lower activation and frequency of effector memory cells. No clear signs of T-cell immunosenescence were observed. NK cells from CFS individuals displayed higher expression of NKp46 and CD69 but lower expression of CD25 in all NK subsets defined. Overall, T cell and NK cell features clearly clustered CFS individuals.

**Conclusions:**

Our findings suggest that alterations in T-cell phenotype and proliferative response along with the specific signature of NK cell phenotype may be useful to identify CFS individuals. The striking down modulation of T cell mediated immunity may help to understand intercurrent viral infections in CFS.

## Background

Chronic Fatigue Syndrome (CFS) is a complex clinical condition of unknown etiology, characterized by persistent or intermittent fatigue that is not the result of recent exertion and does not improve with rest, resulting in a significant reduction in the patient's previous normal activity [1]. Classical diagnostic criteria for CFS overlap with Myalgic Encephalomyelitis (ME) and require these symptoms to be present for at least six months and concomitant to at least four accompanying symptoms, among them: impaired memory, adenopathy, myalgia or polyartralgia [2]. The World Health Organization acknowledged ME/CFS as a disease of the nervous system (ICD G93.3) [3]. However, CFS is a multi-system disease, in which neurological disorders are accompanied by altered immune, musculoskeletal, endocrine and cardiovascular systems [4,5]. Research efforts have recently provided a new International Consensus Criteria for ME [3]. However, the search for the etiology(ies) and the pathogenic mechanisms of CFS has unsuccessfully reviewed several viral hypotheses including herpesviruses or retroviruses as potential triggers of the disease [6-8]. The potential role of pathogens in the CFS field has boosted research in the immunological sides of the illness [9-11]. Indeed, the link between the immune system and CFS has been explored since the early 90’s [12], and is supported by the coincidence of the onset of symptoms with viral infections [13], the persistence of several infections in CFS individuals [10,14-17], the beneficial effect of treatment of human herpesvirus 6 and Epstein-Bar virus infection in CFS symptoms [18] and the reported role of autoimmunity [19,20] Moreover, other features of ME/CFS, such as mild inflammation, oxidative/nitrosative stress, mitochondrial dysfunction and the presence of autoimmunne responses [4] may alter immune function and phenotype.

However, the characterization of the immune status of CFS individuals has frequently yield contradictory results. Early work associated CFS with a general status of immune activation assessed by CD38 or HLA-DR expression in CD8 T cells [12]. However, other authors found similar expression of these markers in CFS and healthy individuals [21], while lower expression of the activation markers CD69 or soluble CD26 has also been described as a feature of CFS [22,23]. Similarly, B-cell function [24], B-cell mediated autoimmunity or unbalanced cytokine network have been linked to CFS [9,25-27] again with inconclusive results. Altered numbers of NK subsets, defined by CD56 or CD16 expression, and an impaired NK-cell lytic activity have been more consistently associated with CFS [24,28-32], although controversial data have been also reported [33] and a consensus in relevant NK cell markers is still lacking.

At least some of these immune features described in CFS may be related to active, poorly controlled viral infections, which differently modulate immune responses and may produce immune hyperactivation/exhaustion, as widely reported for HIV [34]; may contribute to immunosenescence, as postulated for CMV [35]; or may cause a status of immune anergy, as described for measles virus [36]. In this context, we sought to compare the phenotype and function of different immune cells between healthy and ME/CFS individuals. The study was designed in 2010 and could not include new definitions that currently allow for the separation of ME and CFS affected individuals [3]. However, our data point to an unaffected B-cell compartment, a biased NK-cell phenotype and a poorly responsive T-cell compartment as the main immune features of ME/CFS affected individuals.

## Methods

### Patients

A study to screen immunological features of patients suffering from CFS was designed and approved by the Ethics Committee of the Hospital Universitari Germans Trias i Pujol (Barcelona, Spain; EO-10-007). All procedures followed the Helsinki Declaration in 1975. The main objective was to compare phenotypic and functional alterations in immune cells between CFS patients and healthy donors. Therefore, a wide range of phenotypic features was analyzed in a limited number of CFS patients (N = 22) and controls (N = 30). CFS patients fulfilled the Fukuda criteria [1]. Individuals were selected from cohorts of CFS Clinical Units (CFS Unit, Tarragona, Spain and Vall d’Hebron University Hospital, Barcelona, Spain). General exclusion criteria from these cohorts were diabetes, hypertension, chronic obstructive pulmonary disease, inflammatory bowel or Crohn's disease, rheumatoid arthritis, Parkinson or Huntington disorders, schizophrenia, organic mental disorders, substance use disorders, multiple sclerosis and body mnass index BMI > 30 kg/m^2^. Eligible subjects were those with age over 18 years, confirmed diagnosis of CFS for more than two years and absence of current identified infections. Additional exclusion criteria were pregnancy and chemotherapy treatment. In order to mirror the potential heterogeneity of CFS-affected, other major comorbidities associated to CFS were evaluated but were not considered exclusion criteria. In particular, anxiety that is an common feature of CFS affected individuals was evaluated using HADS (Hospital Anxiety and Depression Scale) [37]. The severity of CFS was assessed using a national scale [38]. Informed consent was obtained from all participants in the study. Clinical and demographic data were collected from medical records.

### Sample processing

A single blood sample was collected by venipuncture in EDTA vacutainer tubes (BD Biosciences) from all participants. An aliquot was used for immediate immunophenotype; remaining blood was processed for plasma and peripheral blood mononuclear cells (PBMC) preparation by standard methods as described [39]. PBMC were washed twice in PBS and resuspended in RPMI culture medium for immediate analysis of proliferation, NK cell activity or cell death assays. All samples were collected and freshly processed at the Hospital Germans Trias i Pujol. All samples were processed for immunophenotype and function on the same day of blood collection, no more than four hours were left between sample collection and immunophenotype staining, while no more than six hours were left for functional and cell death assays.

### Determination of absolute counts of B, T and NK cells

Absolute counts of B, T and NK cells were analyzed by flow cytometry. First, the absolute lymphocyte count was determined using an anti-CD45–V450 antibody (BD Biosciences) in combination with perfect-count microspheres (Cytognos). Then, the percentage of the different lymphocyte subsets was determined using the following antibody combination: CD45–V450, CD19–AmCyan, CD3–APC-Cy7, CD4–APC, CD8–PerCP, CD56–PE and CD16–FITC (BD Biosciences). Absolute count of each cellular population was calculated as follows: (X*Y)/100, where X is the percentage of each subset and Y is the absolute count of lymphocytes.

### Immunophenotype

Freshly obtained blood was incubated for 15 minutes at room temperature with the antibody combinations shown in Table 1 in order to characterize B, T and NK cell populations. All antibodies were from BD Biosciences unless indicated. Cells were then lysed for 15 minutes at room temperature in FACS Lysing solution (BD Biosciences), washed in PBS and fixed in PBS containing 1% formaldehyde (Sigma), before acquisition in an LSRII flow cytometer (BD Biosciences). For Ki67 and FOXp3 staining, two aliquots of fresh blood were incubated with extracellular antibodies (Table 1) as indicated above. After lysis, cells were washed and fixed/permeabilized using FOXp3 staining buffer set (eBiosciences) and incubated with anti Ki67 and FOXP3 antibodies or IgG isotype control antibodies (Table 1). Treg were defined by the presence of a separate population of CD25^bright^ FOXP3^+^ cells.

**Table 1:** Antibody combinations used for the phenotypic characterization of T, NK, B cells and for the determination of absolute counts

**Antibody combinations/**							**Fluorochrome**			
**CELL TYPE**	**Phenotype**	**Tube #**	**V450/Pacific blue**	**V500/Am Cyan**	**FITC**	**PE**	**APC/Alexa Fluor 647**	**PerCP/PerCP-Cy5**	**PE-Cy7**	**APC-cy7**
**T cells**	Thymic output/Exhaustion	**T1**			CD95	PD-1	CD4	HLA-DR	CD8	CD3
		**T2**			CD45RA	CD31	CD4	CD38^d^	CD8	CD3
		**T3**					CD4		CD8	CD3
	CD8 activation	**T4**	CD4		HLA-DR		CD45RO	CD38^d^	CD8	CD3
		**T5**	CD4						CD8	CD3
	Main subsets/senescence	**T6**	CD45RA	CD8	CD57^e^	CD28	CD27	CD4	CCR7	CD3
		**T7**		CD8				CD4		CD3
	Treg/Prolif./Anergy	**T8**	CD4	CD8	KI67	FOXP3^e^	CD127^c^	CD5^d^	CD25	CD3
		**T9**	CD4	CD8	IRR IgG	IRR IGG2a^e^				CD3
**NK cells**	Activation/receptor profile	**NK1**	CD69	CD3^b^/CD19^b^	CD107a	Nkp44	CD57^e^	CD25	CD56	CD16
		**NK2**	NKp46^a,ed^	CD3^b^/CD19^b^	CD107a	NKG2D^e^	Nkp30 ^c^	NKG2A^e^	CD56	CD16
		**NK3**		CD3^b^/CD19^b^					CD56	CD16
**B cells**	Mature/Memory/Transitional	**B1**			CD1c	IgD^e^	IgM	CD38	CD27^e^	CD19
		**B2**			CD23	IgD^e^	CD5	CD38	CD10^e^	CD19
		**B3**				IgD^e^				CD19
	Switched memory	**B4**			CD1c^e^	IgG^e^	IgA	CD38	CD27^e^	CD19
		**B5**				IgG^e^	IgA^e^			CD19

^a^ Pacific blue-coupled antibody.

^b^ AmCyan-coupled antibody.

^c^ Alexa Fluor 647-coupled antibody.

^d^ PerCP-Cy5.5 coupled antibody.

^e^ sources different from BD Biosciences (NKp46 and CD57 from BioLegend; NKG2A from R&D Systems, NKG2D and CD1c from Santa Cruz; FOXP3, IRR IGG2a, CD27 and CD10 from eBiosciences; IgG and IgA from Jackson ImmunoResearch, CD57 from Beckman Coulter).

### B-cell and T-cell proliferation assays

Freshly obtained PBMC were stained with 0.33 μM CFSE (Invitrogen) for 5 minutes at room temperature. After extensive washes, cells were cultured in RPMI1640 medium supplemented with 10% of FBS (R10 medium) and different stimulus. The proliferation of T cells was assayed using 5 μg/mL of PHA (Sigma-Aldrich) plus 10 U/mL of IL-2 (Roche). For B cells, 3 μg/mL of endotoxin-free CpG2006 (InvivoGen) or 1 μg/mL of R848 (Alexis Biochemicals) were used alone or in combination with 5 μg/mL of F(ab)2 Goat anti-human Igs (Jackson Immunoresearch). Four days later, cellular proliferation was assessed by flow cytometry after staining T cells with anti CD2–PerCP-Cy5.5, CD5–APC, CD4–V450, CD8–APC-Cy7 and CD19–PE-Cy7 or B cells with CD3–APC-Cy7, CD19–PE-Cy7, CD14–PerCP (BD Biosciences) and IgD-APC (Miltenyi Biotec). Data analysis was performed with the Flowjo software (Tree Star, Inc.), calculating division and proliferation indexes for each condition using best fits provided by the software.

### Cell death assays

Cell death was evaluated by culturing PBMC in of R10 medium at a density of 1 × 10^6^ PBMC/ml for 24 hours [34,40]. For T-cell death analysis, PBMC were incubated with 40 nM of the potentiometric mitochondrial probe DIOC_6_ (Invitrogen), 5 μg/mL propidium iodide (Sigma), and CD3–APC-Cy7, CD4–APC and CD8–PE-Cy7 antibodies. For B cell death analysis, PBMC were incubated with 40 nM of DIOC_6_, 0.3 μM of Sytox Blue (Invitrogen) and CD19–APC-Cy7, IgD–PE, CD38–PerCP-Cy5.5, CD5–APC (BD Biosciences) and CD27–PE-Cy7 (eBiosciences). Cells were acquired in an LSRII flow cytometer; dead cells were identified by their low DIOC_6_ staining [41].

### NK activity assays

Lytic activity of NK cells was assessed as described [42] with slight modifications. Available samples of PBMC (n = 16) were extensively washed to remove any trace of EDTA from extraction tubes and monocytes/macrophages were removed by plastic adherence for 1 h at 37°C (5% CO_2_), this treatment removed 82.4% of monocytes from PBMC samples (n = 13). Exponentially growing eGFP-K562 cells å(obtained through the AIDS Research and Reference Reagent Program, from Dr. Kantakamalakul [43]) were used as target cells. Incubations were performed in duplicate in 96 well plates by seeding a fixed amount (10,000 target cells) alone or with increasing amounts of monocyte-depleted PBMC in R10 medium, covering a range from 80:1 to 2.5:1 effector:target cell ratios. Samples were incubated for 4 h at 37°C (5% CO_2_) stained for 10 min with 5 μg/mL Propidium Iodide (PI, Sigma) and acquired in a LSRII flow cytometer. The percentage of dead (PI^+^) eGFP-K652 cells in the gate of eGFP-K562 cells was calculated.

### Clustering and statistical analyses

Continuous variables were expressed as the median (interquartile range) and compared using the Mann–Whitney non-parametric test. Discrete variables were described as percentages (number of patients) and the chi-square or Fisher exact test was used as appropriate. P values <0.05 were considered significant. Clustering of CFS and healthy individuals was performed using the Cluster 3.0 software. Data were normalized according to medians and clustered using non-parametric correlations. Treeview 1.1 software was used to generate and visualize dendrograms.

## Results

### Patient characteristics

The main characteristics of the individuals recruited for the study are summarized in Table 2. Both Control and CFS groups showed similar median age values (38 and 44 years, respectively, *p* = ns, Mann–Whitney test) and were mostly composed by females (55% and 73% respectively, *p* = ns, Fisher exact test). Patients suffering from CFS showed a median [IQR] time from certified diagnosis of 3 [3-5] years, with 53% of them reporting onset of symptoms with viral infections. The median grade of CFS assessed by a national scale [38] was 3, while only three recruited individuals showed grade 2. Grade 3 was comparable to mild level of severity (an approximate 50% reduction in pre-illness activity level) according to the newest consensus criteria for ME [3]. Grade 2 is assignable to a moderate severity. No severe cases were included in the study. Three individuals in the CFS group were excluded from the analysis, one due to a B cell lymphocytosis (B cells represented 24% of lymphocytes showing a total B cell count of 435 cells/μL, with more than 85% of cells showing a IgD^+^IgM^–^CD23^+^CD27^+^CD5^+^CD38^–^ phenotype), one due to an IgA deficiency and a third due to sample unavailability.

**Table 2:** Main characteristics of individuals recruited in the study

	**SFC**	**HD**	***P-value***
	**(N = 22)**	**(N = 30)**	**(Mann–Whitney)**
**Age** (years, Median, IQR)	44 [40–50]	38 [33–52]	*ns*
**Gender** (% of female)	73	55	*ns*
**Time from diagnosis** (Years, Median, IQR)	3 [3–5]	-	
**Grade of Fatigue** (Median, IQR)	3 [2–3]	-	
**Reported onset with viral infection** (%)	53	-	

Additional factors that may modulate immune function such as comorbidities and polypharmacy associated with CFS were also evaluated. Polypharmacy is described in Table 3. The main potential interference of pharmacy was homeopathy (52% of patients), antioxidants (52%) and analgesics (42%, mainly paracetamol), which were separately distributed among participants.. Regarding comorbidities, anxiety was the most prevalent; myalgia or tendinopathy along with reported multiple chemical sensitivity were also relevant (Additional file 1: Table S1).

**Table 3:** Self-reported polypharmacy in CFS affected individuals

	**Analgesics**			**Antidepressants Anxiolytics Benzodiazepines**			**Homeopa Thy**	**Antioxidants**
**Patient #**	**Paracetamol**	**Ibuprofen**	**Tramadol**	**Duloxetine**	**Pregabalin**	**Diazepam**		
CFS1	**-**	**-**	**-**	**-**	**-**	**-**		
CFS2	**-**	**-**	**-**	**-**	**-**	**-**	**+**	**+**
CFS3	**-**	**-**	**-**	**+**	**-**	**-**	**+**	**+**
CFS4	**-**	**-**	**-**	**-**	**-**	**+**	**+**	**+**
CFS5	**-**	**-**	**-**	**-**	**-**	**-**	**+**	**+**
CFS6	**-**	**-**	**-**	**-**	**-**	**-**	**+**	**+**
CFS7	**-**	**-**	**-**	**-**	**-**	**-**	**+**	**+**
CFS8	**-**	**-**	**-**	**-**	**-**	**-**	**+**	**+**
CFS9	**-**	**-**	**-**	**-**	**-**	**-**	**+**	**+**
CFS10	**-**	**-**	**-**	**-**	**-**	**+**	**+**	**+**
CFS11	**-**	**-**	**-**	**-**	**-**	**-**	**-**	**+**
CFS12	**+**	**-**	**+**	**-**	**+**	**-**	**-**	**-**
CFS13	**-**	**-**	**-**	**-**	**-**	**-**	**-**	**-**
CFS14	**+**	**-**	**-**	**-**	**-**	**-**	**-**	**-**
CFS15	**+**	**+**	**-**	**+**	**+**	**-**	**-**	**-**
CFS16	**+**	**+**	**-**	**+**	**+**	**-**	**-**	**-**
CFS17	**+**	**-**	**-**	**-**	**-**	**+**	**-**	**-**
CFS18	**+**	**+**	**-**	**+**	**+**	**+**	**-**	**-**
CFS19	**-**	**-**	**-**	**-**	**-**	**-**	**-**	**-**

Daily antioxidant doses were: 500 mg omega-3 fatty acids (Docosahexaenoic acid DHA/Eicosapentaenoic acid EPA), 150 mg Methylsulfonylmethane, 150 mg alpha lipoic àcid, 75 mg Superoxide dismutase (SOD), 50 mg N-Acetyl cisteine (NAC), 3,5 mg Zn2+ and 0.5 mg Cu2 + .

### Quantification of main lymphocyte subsets

**Figure 1:**
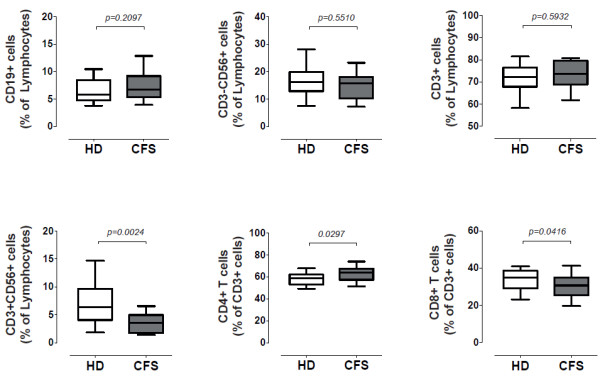
**Analysis of major lymphocyte subsets in CFS affected individuals.** Fresh blood was stained with anti CD45, CD19, CD3, CD4, CD8, CD16 and CD56 antibodies. The percentage of NK (CD3^–^CD56^+^), B (CD19^+^) and T cells (CD3^+^) was analyzed in gated CD45^+^ lymphocytes. Similarly, after gating CD3^+^ lymphocytes the percentage of CD4^+^, CD8^+^ or CD56^+^ cells was analyzed. Figures show data from healthy donors (n = 24, HD) and SFC affected individuals (n = 17, SFC) with median values (lines), interquartile ranges (boxes) and 10–90 percentile values (bars). In all cases, *p*-values for nonparametric Mann–Whitney comparison are shown.

The absolute numbers and percentages of B cells (CD19^+^), NK cells (CD3-CD56^+^CD16^+^), T cells (CD3^+^), CD4 T cells (CD3^+^CD4^+^) and CD8 T cells (CD3^+^CD8^+^) in the CD45^+^ lymphocyte gate were similar between CFS individuals and healthy donors (data not shown and Figure 1). However, significantly lower frequency of CD3^+^CD56^+^ lymphocytes was observed in the CFS group. Moreover, a slightly unbalanced composition of the CD3^+^ cell subset was also observed, showing a higher CD4 T-cell representation in the CFS group (Figure 1).

### B-cell phenotype and function

The potential role of B cells on CFS has been reinforced by recent data on the clinical benefit of Rituximab treatment [19,20]. Thus, we characterized circulating B cells using the antibody panels shown in Table 1. No significant differences between groups were observed in the percentage of IgD^+^, IgG^+^, IgA^+^ or CD27^+^ B cells, indicating a similar memory compartment in CFS and control individuals. Moreover, the levels of transitional (CD19^+^IgD^+^CD38^high^CD10^+^CD5^+^), plasma-plasmablastic (CD19^+^CD27^high^CD38^high^) or marginal zone B cells (CD19^+^IgD^+^IgM^+^CD27^+^CD1c^+^), were comparable in both groups (Additional file 1: Figure S1). Consistently, functional assessment of B-cell responses showed similar proliferation to different stimuli targeting TLR9 and TLR7/8, and similar level of *ex vivo* cell death (Additional file 1: Figure S1 and data not shown). Thus, no major perturbations on the phenotype and function of circulating B cells could be identified.

### NK-cell phenotype and function

**Figure 2:**
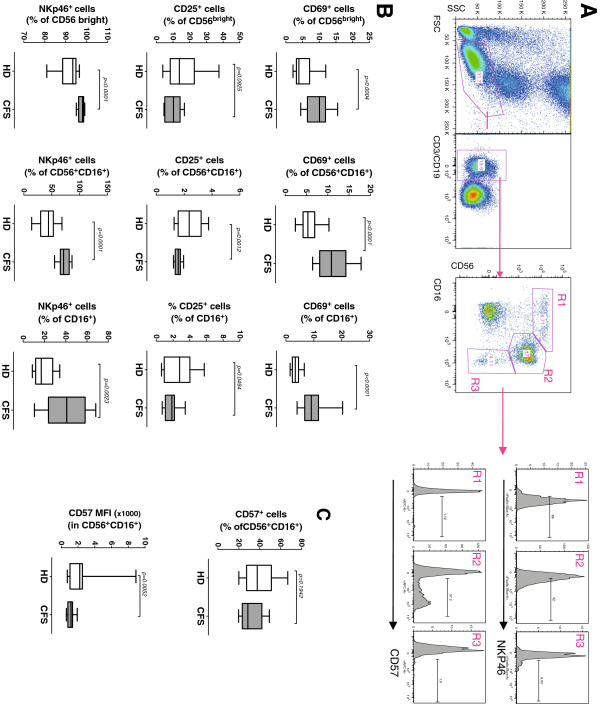
**Analysis of NK cell phenotype in CFS affected individuals.** Fresh blood was stained with the antibody combinations described in Table 1. Panel **A.** NK cells were gated as CD3^-^CD19^-^ PBMC and analyzed for CD16 and CD56 staining defining CD56 bright (R1), CD56^+^CD16^+^ (R2) or CD16^+^ (R3) gates. Representative histograms showing the expression of NKp46 (upper plots) and CD57 (lower plots) are shown. Panel **B**. NK cell subsets gated according to Panel A were analyzed for the expression of CD69 (upper), CD25 (middle) and NKp46 receptor is shown. Panel **C.** In parallel, double positive CD56^+^CD16^+^ NK cells were analyzed for the expression of CD57, as the percentage of positive cells (upper graph) or the Mean Fluorescence intensity (lower graph). In all cases, data from healthy donors (n = 25, HD) and SFC affected individuals (n = 19, SFC) are shown, with median (thick lines), interquartile range (boxes) and 10–90 percentile values (bars). In all cases, *p*-values for nonparametric Mann–Whitney comparison are shown.

NK-cell alterations have been classically associated with CFS, showing decreased numbers and function [9,44]. Therefore, we evaluated the phenotype of NK cells using the antibody panel shown in Table 1. The three main NK-cell subsets identified in our gating strategy CD56^high^CD16^–^, CD56^+^CD16^+^ and CD16^+^CD56^–^ cells (Figure 2A) and most of the markers analyzed were comparable between groups (data not shown). However, the expression of CD69 and NKp46 was significantly higher in CFS individuals, while the expression of CD25, was significantly lower (Figure 2B).

A phenotypic feature of NK cells from CFS individuals is the low expression of CD57 [45]. Figure 2C shows that in our study, the percentage of CD57 expressing NK cells is similar among groups, although the intensity of CD57 staining showed significantly lower values in CFS individuals. Despite alterations in phenotype, NK cell cytotoxic activity did not reach significant differences between groups in a subset of samples (9 CFS and 7 HD) available for functional assays. Furthermore, no differences in sensitivity of NK cells to *ex vivo* cell death could be detected between groups (data not shown).

### T-cell phenotype and function

**Figure 3:**
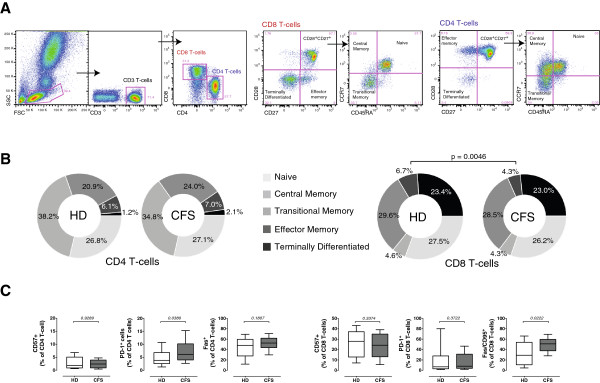
**Analysis of CD4 and CD8 T cell subsets, immunosenescence and exhaustion.** Panel **A.** Fresh blood was stained with the antibody combinations described in Table 1. Different CD4 and CD8 T cell subpopulations (Naïve, Central memory, Transitional memory and Effector memory) were identified by CD27, CD27 and then CCR7 and CD45RA expression as shown. Panel **B**. The median values for the frequency of the indicated subsets in healthy donors and CFS affected individuals are shown in circular plots. Significant differences among groups are indicated. Panel **C.** The entire CD4 and CD8 T cell gates were also analyzed for the expression of CD57, PD-1 and Fas-CD95. In all cases, data from healthy donors (n = 25, HD) and SFC affected individuals (n = 19, SFC) are shown, with median (thick lines), interquartile range (boxes) and 10–90 percentile values (bars). In all cases, *p*-values for nonparametric Mann–Whitney comparison are shown.

Several authors have pointed to a general status of T-cell activation in CFS [12] that may be consistent with intercurrent viral infections. A similar scenario has been described for HIV infection, in which chronic viral infection alters the balance of naïve, central and effector memory cells [46], and increases hyperactivation, immunosenescence and apoptosis [34,47,48]. In our cohort, CD4 T cells from CFS individuals and controls showed similar levels of naïve (CD45RA^+^CCR7^+^CD27^+^CD28^+^), central (CD45RA^–^CCR7^+^CD27^+^CD28^+^), transitional (CD45RA^–^CCR7^-^CD27^+^CD28^+^), effector (CD45RA^–^CCR7^-^CD27^+^CD28^-^) and terminally differentiated memory (CD45RA^-^CCR7^-^CD27^-^CD28^-^) cells (Figure 3A and B). Most CD8 T cell subsets were also similar in both groups, although CFS affected individuals showed lower frequency of cells with an effector phenotype (defined in CD8 T cells by the following markers CD45RA^-^CCR7^-^CD27^-^CD28^+^, Figure 3B). This observation maybe related with the lower level of CD56 expression in CD3^+^ cells (Figure 1), although the heterogeneity of this latter population impedes a proper interpretation of these data. We also analyzed several markers of immunosenescence or immune exhaustion, with discordant results: CFS and control individuals had similar expression of the T-cell immunosenescence marker CD57 [46] in CD4 and CD8 T cells, while differences were observed in the expression of exhaustion markers PD-1 and CD95 [46] in CD4 and CD8 T cells, respectively (Figure 3C).

**Figure 4:**
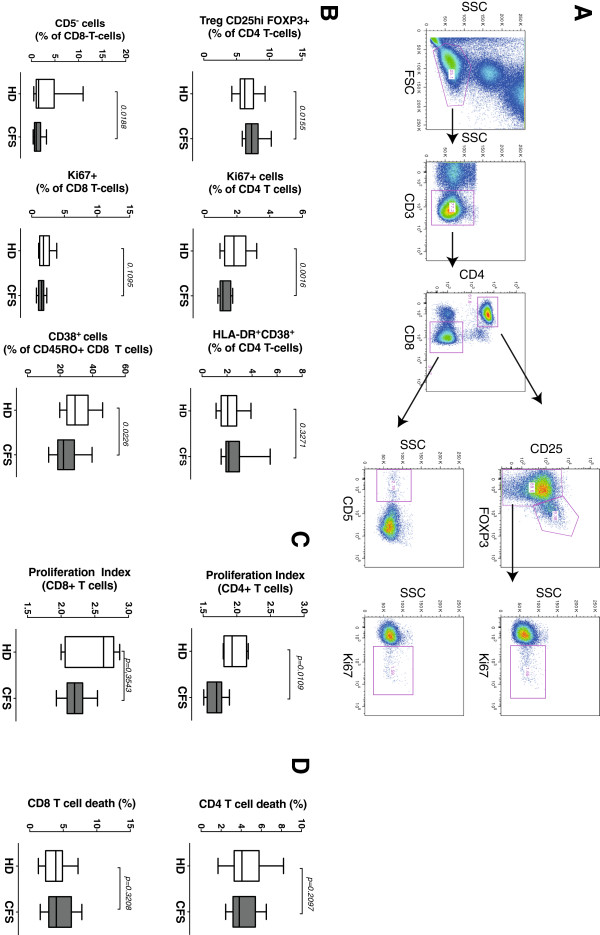
**Analysis of CD4 and CD8 T cell activation, proliferative capacity and death.** Panel **A.** Gating strategy to analyze FOXP3 and Ki67 expression. CD4 and CD8 T cells were identified in a CD3+ gate. In CD4 T cells, the expression of FOXP3 and CD25 defined the Treg population, while remaining cells were analyzed for Ki67 expression. In CD8 T cells, CD5 and Ki67 were analyzed in the whole population. Panel **B.** The entire CD4 and CD8 T cell gates illustrated in Figure 3 were analyzed for the frequency of Treg (CD25^+^FOX^–^P3^+^) cells, or for the expression of the indicated markers. In all cases, data from healthy donors (n = 25, HD) and SFC affected individuals (n = 19, SFC) are shown. Panel **C.** PBMC from healthy donors (n = 5, HD) and CFS affected individuals (n = 8, CFS) were stained with CFSE and cultured in the presence of a combination of PHA and IL-2 (PHA/IL-2). Data shown are proliferation index of gated CD4 or CD8 T cells calculated from best-fit curves using FlowJo software. Panel **D.** PBMC from healthy donors (n = 30, HD) and CFS affected individuals (n = 19, CFS) were also cultured for 24 h to assess spontaneous CD4 or CD8 T-cell death using DIOC_6_ and PI staining. Results show total cell death defined by low DIOC_6_ fluorescence signal. In all panels, median values (thick lines), interquartile ranges (boxes) and 10–90 percentile values (bars). In all cases, *p*-values for nonparametric Mann–Whitney comparison are shown.

We assessed the frequency of T regulatory cells (Treg) and several proliferation/activation markers (Figure 4A). Treg cells defined as CD4^+^CD25^++^FOXP3^+^ or CD4^+^CD25^++^FOXP3^+^CD127^–^ showed significantly higher percentages in CFS individuals (Figure 4B and data not shown), concomitant with lower levels of Ki67^+^ cells in CD4 T cells (Figure 4B). In contrast, CD8 T cells did not show differences among groups in Ki67 positivity, although CFS individuals displayed higher expression of CD5 (Figure 4B), a marker associated with impaired T-cell responses [49,50]. CFS group also showed lower levels of the activation marker CD38 in total and the memory (CD45RO^+^) CD8 T cells (not shown and Figure 4B). Despite these small differences in T-cell subsets, *ex vivo* proliferative responses of CD4 T cells were significantly lower in CFS individuals although showed no differences among groups for CD8 T cells (Figure 4C). Finally, no differences in cell death were noticed for CD4 and CD8 T cells among groups (Figure 4D).

As a whole, these data suggest an impaired T-cell response in CFS individuals associated with increased Treg numbers and with some specific markers in both CD4 and CD8 T cells, but not directly caused by a general status of immunosenescence.

### Analysis of T-cell and NK-cell phenotype as markers for CFS

**Figure 5:**
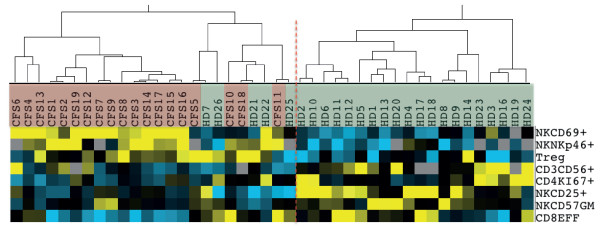
**Clustering CFS individuals according to NK and T cell phenotypic markers.** A subset of 19 CFS (red labels) and 25 control individuals (green labels) was analyzed. Figure shows normalized centered data in yellow (for positive values, above median) and blue (for negative values, below median). Two groups of CFS and Control individuals were clearly differentiated, while a heterogeneous subgroup was clustered with CFS individuals.

In order to evaluate the potential utility of the described immunological markers for the identification of CFS cases, an unsupervised clustering analysis was performed. While unrestricted selection of markers provided poor ability to separate CFS individuals and HD, marker selection according to their selectivity yielded a combination of 8 NK and T-cell phenotypic parameters that showed the best resolution in classifying CFS and healthy individuals (*p* = 3.3×10^-8^, Figure 5). The NK markers: CD25, CD69, NKp46 (expressed as percentage of positive cells) and CD57 (as fluorescence intensity) in combination with the T-cell subsets: regulatory, proliferating Ki67^+^CD4^+^, effector CD8^+^ and CD56^+^ T cells, showed a high sensitivity (100%) but a moderate false positive rate (5/24, specificity 79%, Figure 5). A more restrictive choice of parameters, including exclusively NK cell markers showed similar false positive rates (5/23, specificity 78%) but lower sensitivity (95%) in the detection of CFS cases (Additional file 1: Figure S2).

## Discussion

The immunological status of individuals suffering from CFS has yielded heterogeneous results [12,17,31,51,52]. Clear examples are data from different CFS cohorts analyzed in a single laboratory [32]. These observations may be the consequence of an intrinsic heterogeneity in the classification of CFS affected individuals [3], or could be related to the presence of intercurrent infections in the individuals recruited in the different studies. In particular, the prevalence of infections by herpesviruses or enteroviruses [15], which have been described more frequently in CFS individuals [18,53], are known to modulate immune phenotype [46]. We have performed a wide screening of the phenotype and function of B, NK and T cells in CFS. In contrast to other studies, our main inclusion criterion was focused on the lack of active infections, rather than on the CFS-related comorbidities. Although this could be a limitation, as comorbidities may also affect immune status, we believed that these criteria might provide a more homogeneous immune profile of CFS.

Our data suggest that most differences between CFS and healthy controls were observed in NK and T cells; while the B-cell compartment showed similar composition in both groups. Importantly, these differences could not be associated to polypharmacy or comorbidities (Additional file 1: Figure S3 and data not shown), although the sample size of our study limited the statistical power of these analyses. The lack of B cell alterations observed in our study contrasts with the active role of B cells and autoimmune responses in CFS that have been highlighted by the recent clinical use of Rituximab [19]. The possibility that B-cell alterations are restricted to tissue B cells may explain this apparent contradiction. However, it should be noted that in contrast to NK and T cell markers described herein, parameters of B cell phenotype showed heterogeneous values and seem to be more affected by antioxidant/analgesic treatments. This could be a second limitation of our study; thus, a more detailed analysis of B cell phenotype and function using larger cohorts will be required to fully understand the role of these cells in CFS.

A different scenario is observed in NK cells. In this case, several markers showed consistent alterations in CFS individuals and illustrate a skewed NK cell population with high CD69 and low CD25 expression, a paradoxical phenotype that has been described in acute influenza infection or vaccination [54] and that is in clear conflict with recent data reporting low CD69 expression in NK and T cells from CFS affected individuals [22,23]. Of note, this latter work measured CD69 expression done after in vitro stimulation while our data were obtained using freshly obtained unstimulated cells. Thus, although apparently in conflict, both data may reflect different aspects of a deregulated CD69 expression in CFS. We have also observed increased levels of NKp46 expression in CFS individuals. Interestingly the expression of this receptor has been recently linked to T-cell responses in murine models [55]; however, no clear association could be found in our cohort. The phenotype of NK cells is controlled by genetic, epigenetic and environmental factors and probably summarizes the infectious history of an individual in a poorly-understood form of immunological memory [56]. Specific phenotypes have been associated to infectious agents, such as CMV or influenza [54,57]. Although it could be tempting to speculate on a common infective history in our cohort of CFS individuals, the analysis of their serological status for some viruses (CMV, EBV, parvoviruses) did not show a clear link between past infections and current NK-cell phenotype. Probably, wider studies are required to answer this relevant question and to confirm the association of NK-cell phenotype with impaired lytic activity, which has been described in larger cohorts but failed to reach significant differences in our limited analysis, probably due to the small subset of samples analyzed or to the use of EDTA as anticoagulant. NK function decreases with age, a phenomenon associated with NK immunosenescence that can be evidenced by the increased expression of CD57, present in terminally differentiated NK cells [58]. However, there is no consensus on the role of CD57 in immunosenescence of NK cells and CFS has been associated with low expression of CD57 [59]; an observation partly confirmed in our cohort.

The most unanticipated data in our cohort of CFS individuals is related to T-cell phenotype and function, which could be defined as a general hyporesponsiveness. These data contrast with descriptions of high T-cell activation in CFS [12], but are consistent with other reports describing reduced CD8 cytotoxic activity [9]. Again, the presence of active viral infections at the sampling time may be a source of heterogeneity. Alternatively, the leakage of bacterial products from gut may also determine T-cell activation [60,61]. To explore this possibility, we analyzed plasma levels of sCD14 In healthy and CFS individuals, showing similar median levels (4.5 and 4.7 μg/ml, respectively, *p* = 0.44, data not shown), suggesting that gut leakage is not a major contributor to immune alterations in our cohort.

Our data may suggest a general default of T-cell function that can be observed using different makers in both CD4 and CD8 subsets. While CD4 T cells show lower Ki67 staining and *ex vivo* proliferation, CD8 T cells showed no proliferative differences, but lower levels of CD56 expression, effector (CCR7^–^CD45RA^–^) cells, CD38^+^ cells and higher expression of CD5, a marker of anergy associated to continuous antigen exposure [50]. Furthermore, this general status of T-cell hyporesponsiveness seems to be unrelated to immunosenescence, since no differences in CD57 or no increased levels of CD27^-^ or CD28^-^ cells were observed between groups. A key factor in the control of T cell responses is the function of Treg cells [62], which are significantly increased in our cohort of CFS individuals confirming data from another recent study [9]. Although no clear correlation was observed between Treg frequency and other markers of NK cell or T-cell phenotype; an active role of Treg could be supported by reported data on key mediators of Treg action, such as TGF-β [63], that seem to be also upregulated in CFS individuals [64,65].

The potential use of the immunological markers identified in this study was explored in cluster analyses. While the best resolution required both T and NK cell markers, we also identified a robust combination of NK cell markers that may be useful for diagnosis. This combination includes CD25, CD69 and NKp46 expression in CD56 + CD16+ cells and CD56 expression in CD3^+^ cells, suggesting that a five-color flow cytometry strategy including these markers may be useful for diagnostic purposes. However, a wider range of parameters including FOXP3 and Ki67 expression in CD4 T cells improved specificity. The use of these potential combinations as diagnostic tools requires validation in further studies, including both larger cohorts and a wider range of CFS clinical status.

In conclusion, CFS individuals analyzed in this study show no differences in B-cell compartment, skewed NK cells and poorly responsive T cells. The observed immunological defaults do not provide any causative link to the illness, but could explain some of the symptoms and in particular the poor control of viral infections reported in these individuals [15,53]. However, some of these markers along with other previously described, such as NK function or DPPIV [22,66] may be useful for the characterization of CFS. However, major roadblocks still exist to reach a reliable combination of immune biomarkers for CFS. First, the potential different etiologies or comorbidities of CFS and second the clear identification of the target population (ME, CFS or both). In addition, immunological markers may reveal only a part or the complex pathogenic spectrum of ME/CFS. Most likely, immune features in combination with a detailed analysis of intercurrent infections and previously described [9,22,25,29,66,67]neurological and metabolic disorders may provide the clues to define a full set of markers helpful for our knowledge of CFS pathogenesis and for its clinical management.

## Competing interest

The authors declare that they have no competing interests.

## Authors’ contribution

MC, JC and MM performed experiments, analyzed data and wrote the manuscript. JR, JA AMG-Q and EN selected patients and edited the manuscript, JCM supervised clinical criteria and edited the manuscript, JP supervised sample extraction and processing, BC, CC and JB designed the study and wrote the manuscript. All authors read and approved the final manuscript.

## Supplementary Material

Additional file 1: Table S1. Main comorbidities identified in CFS affected individuals. **Figure S1.** Analysis of B-cell subsets and proliferation. Panel A. Gating strategy for the identification of B-cell subsets. IgA, IgG and IgD expressing cells were identified in the CD19+ gate, the expression of CD27 in each subset was analyzed. Plasma cells were identified by high CD38 and CD27 expression. Marginal Zone (MZ) B cells by high CD1c expression and transitional cells by CD5, CD10 and CD38 expression. Panel B. Fresh blood was stained with the antibody combinations described in Table I, lysed washed and acquired. B-cell subsets showed similar values in healthy donors (n = 27, HD, empty boxes) and CFS individuals (n = 19, CFS, solid boxes). Panel C. PBMC were stained with CFSE and cultured in the presence of the TLR9 agonist CpG2006 and the TLR7/8 agonist R848. Values of division index calculated using Flow Jo software from healthy donors (n = 5, HD, empty boxes) and CFS individuals (n = 9, CFS, solid boxes) are shown. In all cases, median values, interquartile ranges (boxes), 10-90 percentiles (bars) and p-values for nonparametric Mann-Whitney comparison are shown. **Figure S2.** Clustering CFS individuals according to NK cell phenotypic markers. A subset of 19 CFS (red labels) and 25 control individuals (green labels) was analyzed. Figure shows normalized centered data in yellow (for positive values, above median) and blue (for negative values, below median). NK cell parameters provided lower resolution than the combination of NK and T cell dta. However, CFS and healthy donors showed significant clustering (p = 3.1 × 10-7). **Figure S3.** Analysis of the effect of antioxidant intake on main biomarkers of CFS. 25 control individuals (HD) and 19 CFS individuals subgrouped according to antioxidant treatment were analyzed. Figure shows median and interquartile ranges for the 8 parameters defined in Figure 5. All figures show p-values for 1-way ANOVA analyses of the three groups (upper left corners) and p-values for Mann-Whitney comparisons between the CFS subgroups (right). Click here for file
