# Supplementary material for: Screening NK-, B- and T-cell phenotype and function in patients suffering from Chronic Fatigue Syndrome
Source: J Transl Med. 2013 Mar 20;11:68. doi: 10.1186/1479-5876-11-68 (PMC3614537; doi:10.1186/1479-5876-11-68)
Supplement: Additional file 1: Table S1. — Main comorbidities identified in CFS affected individuals. Figure S1. Analysis of B-cell subsets and proliferation. Panel A. Gating strategy for the identification of B-cell subsets. IgA, IgG and IgD expressing cells were identified in the CD19+ gate, the expression of CD27 in each subset was analyzed. Plasma cells were identified by high CD38 and CD27 expression. Marginal Zone (MZ) B cells by high CD1c expression and transitional cells by CD5, CD10 and CD38 expression. Panel B. Fresh blood was stained with the antibody combinations described in Table I, lysed washed and acquired. B-cell subsets showed similar values in healthy donors (n = 27, HD, empty boxes) and CFS individuals (n = 19, CFS, solid boxes). Panel C. PBMC were stained with CFSE and cultured in the presence of the TLR9 agonist CpG2006 and the TLR7/8 agonist R848. Values of division index calculated using Flow Jo software from healthy donors (n = 5, HD, empty boxes) and CFS individuals (n = 9, CFS, solid boxes) are shown. In all cases, median values, interquartile ranges (boxes), 10-90 percentiles (bars) and p-values for nonparametric Mann-Whitney comparison are shown. Figure S2. Clustering CFS individuals according to NK cell phenotypic markers. A subset of 19 CFS (red labels) and 25 control individuals (green labels) was analyzed. Figure shows normalized centered data in yellow (for positive values, above median) and blue (for negative values, below median). NK cell parameters provided lower resolution than the combination of NK and T cell dta. However, CFS and healthy donors showed significant clustering (p = 3.1 × 10^-7^). Figure S3. Analysis of the effect of antioxidant intake on main biomarkers of CFS. 25 control individuals (HD) and 19 CFS individuals subgrouped according to antioxidant treatment were analyzed. Figure shows median and interquartile ranges for the 8 parameters defined in Figure 5. All figures show p-values for 1-way ANOVA analyses of the three groups (upper left corners) and p-valu [file 1479-5876-11-68-S1.docx]

**Supplementary Table I. Main comorbidities identified in CFS affected individuals.**

| **Patient**  **#** | **FM** | **MPS** | **SS** | **MCS** | **JHS** | **TE** | **RAX** | **RD** |
| --- | --- | --- | --- | --- | --- | --- | --- | --- |
| CFS1 | **-** | **-** | **-** | **-** | **-** | **-** | **+** | **+** |
| CFS2 | **-** | **-** | **-** | **-** | **-** | **-** | **+** | **-** |
| CFS3 | **-** | **+** | **+** | **+** | **-** | **+** | **+** | **-** |
| CFS4 | **-** | **+** | **-** | **+** | **-** | **+** | **+** | **-** |
| CFS5 | **-** | **+** | **-** | **-** | **-** | **-** | **+** | **-** |
| CFS6 | **-** | **+** | **-** | **-** | **-** | **-** | **+** | **-** |
| CFS7 | **-** | **+** | **-** | **+** | **-** | **-** | **+** | **-** |
| CFS8 | **-** | **+** | **-** | **+** | **-** | **+** | **+** | **-** |
| CFS9 | **-** | **+** | **-** | **-** | **-** | **-** | **+** | **-** |
| CFS10 | **-** | **+** | **-** | **-** | **-** | **-** | **-** | **-** |
| CFS11 | **-** | **-** | **-** | **+** | **-** | **+** | **-** | **-** |
| CFS12 | **+** | **+** | **+** | **-** | **+** | **-** | **+** | **-** |
| CFS13 | **-** | **-** | **-** | **+** | **-** | **-** | **-** | **-** |
| CFS14 | **+** | **+** | **+** | **-** | **-** | **+** | **+** | **-** |
| CFS15 | **-** | **+** | **-** | **-** | **-** | **-** | **-** | **-** |
| CFS16 | **-** | **-** | **-** | **-** | **-** | **+** | **+** | **-** |
| CFS17 | **+** | **+** | **+** | **-** | **-** | **-** | **+** | **-** |
| CFS18 | **-** | **+** | **+** | **-** | **+** | **+** | **+** | **-** |
| CFS19 | **-** | **-** | **+** | **-** | **-** | **-** | **-** | **-** |

**Abbreviations:** FM: Fibromyalgia. MPS: Myofascial pain syndrome. SS: Sicca syndrome. MCS: Multiple Chemical sensitivities. JHS: Joint Hypermobility Syndrome. TE: Tendinophaty. RAX: Reactive Anxiety. RD: Reactive Depression.


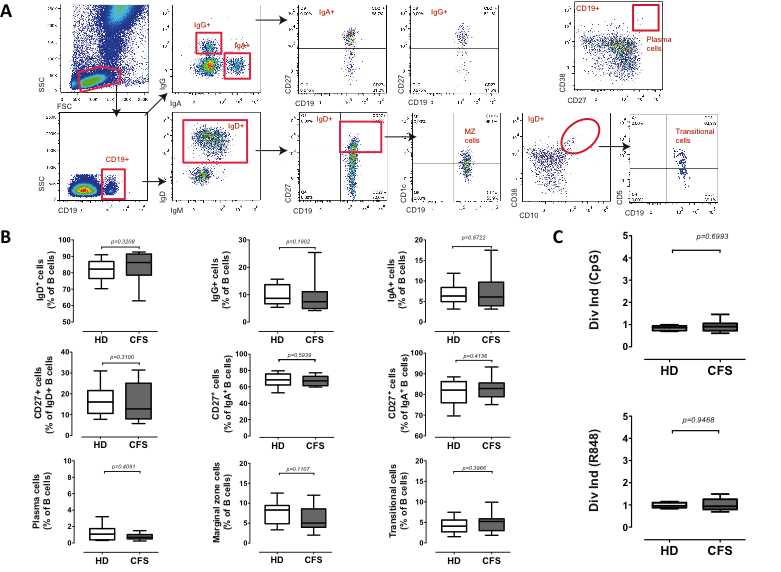


**Supplementary Figure 1. Analysis of B-cell subsets and proliferation.** **Panel A.** Gating strategy for the identification of B-cell subsets. IgA, IgG and IgD expressing cells were identified in the CD19+ gate, the expression of CD27 in each subset was analyzed. Plasma cells were identified by high CD38 and CD27 expression. Marginal Zone (MZ) B cells by high CD1c expression and transitional cells by CD5, CD10 and CD38 expression. **Panel B.** Fresh blood was stained with the antibody combinations described in Table I, lysed washed and acquired. B-cell subsets showed similar values in healthy donors (n=27, HD, empty boxes) and CFS individuals (n=19, CFS, solid boxes). **Panel C.** PBMC were stained with CFSE and cultured in the presence of the TLR9 agonist CpG2006 and the TLR7/8 agonist R848. Values of division index calculated using Flow Jo software from healthy donors (n=5, HD, empty boxes) and CFS individuals (n=9, CFS, solid boxes) are shown. In all cases, median values, interquartile ranges (boxes), 10-90 percentiles (bars) and *p*-values for nonparametric Mann-Whitney comparison are shown.


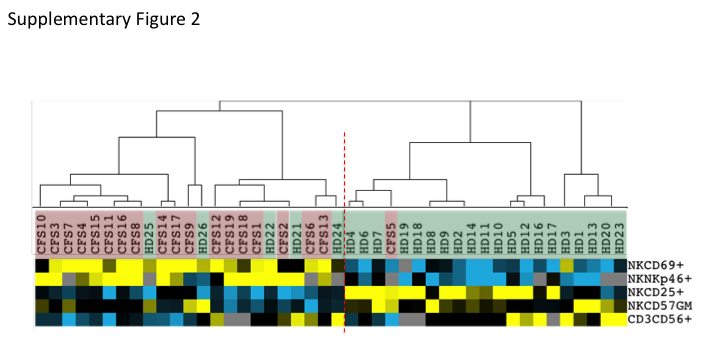


**Suplementary Figure 2. Clustering CFS individuals according to NK cell phenotypic markers.** A subset of 19 CFS (red labels) and 25 control individuals (green labels) was analyzed. Figure shows normalized centered data in yellow (for positive values, above median) and blue (for negative values, below median). NK cell parameters provided lower resolution than the combination of NK and T cell dta. However, CFS and healthy donors showed significant clustering (p=3.1x10-7).


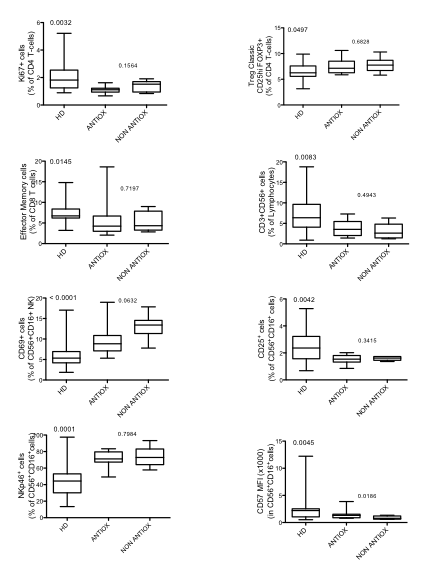
**Suplementary Figure 3. Analysis of the effect of antioxidant intake on main biomarkers of CFS.** 25 control individuals (HD) and 19 CFS individuals subgrouped according to antioxidant treatment were analyzed. Figure shows median and interquartile ranges for the 8 parameters defined in Figure 5. All figures show p-values for 1-way ANOVA analyses of the three groups (upper left corners) and p-values for Mann-Whitney comparisons between the CFS subgroups (right).
